# The role of GPR39 zinc receptor in the modulation of glutamatergic and GABAergic transmission

**DOI:** 10.1007/s43440-023-00478-0

**Published:** 2023-03-30

**Authors:** Gabriela Starowicz, Dominika Siodłak, Gabriel Nowak, Katarzyna Mlyniec

**Affiliations:** 1 Department of Pharmacobiology, Jagiellonian University Medical College, Medyczna 9, 30-688 Krakow, Poland; 2 Laboratory of Trace Elements Neurobiology, Department of Neurobiology, Institute of Pharmacology, Polish Academy of Sciences, Smetna Street 12, 31-343 Krakow, Poland

**Keywords:** GPR39, Glutamate, GABA, Depression, NMDA, Zinc

## Abstract

**Background:**

Despite our poor understanding of the pathophysiology of depression, a growing body of evidence indicates the role of both glutamate and gamma-aminobutyric acid (GABA) signaling behind the effects of rapid-acting antidepressants (RAADs). GPR39 is a zinc-sensing receptor whose activation leads to a prolonged antidepressant-like response in mice. Both GPR39 and zinc can modulate glutamatergic and GABAergic neurotransmission, however, exact molecular mechanisms are still elusive. In this study, we aimed to research the role of glutamatergic and GABAergic system activation in TC-G 1008 antidepressant-like effects and the disruptions in this effect caused by a low-zinc diet.

**Methods:**

In the first part of our study, we investigated the role of joint administration of the GPR39 agonist (TC-G 1008) and ligands of the glutamatergic or GABAergic systems, in antidepressant-like response. To evaluate animal behaviour we used the forced swim test in mice. In the second part of the study, we assessed the effectiveness of TC-G 1008-induced antidepressant-like response in conditions of decreased dietary zinc intake and its molecular underpinning by conducting a Western Blot analysis of selected proteins involved in glutamatergic and GABAergic neurotransmission.

**Results:**

The TC-G 1008-induced effect was blocked by the administration of NMDA or picrotoxin. The joint administration of TC-G 1008 along with muscimol or SCH50911 showed a trend toward decreased immobility time. Zinc-deficient diet resulted in dysregulation of GluN1, PSD95, and KCC2 protein expression.

**Conclusions:**

Our findings indicate the important role of glutamate/GABA signaling in the antidepressant-like effect of TC-G 1008 and imply that GPR39 regulates the balance between excitatory and inhibitory activity in the brain. Thus, we suggest the zinc-sensing receptor be considered an interesting new target for the development of novel antidepressants.

**Supplementary Information:**

The online version contains supplementary material available at 10.1007/s43440-023-00478-0.

## Introduction

Since the discovery of iproniazid and imipramine as the first successful pharmacological treatments for mood disorders in the late 50 s, our understanding of the mechanisms behind the effects of antidepressants have changed several times [1, 2]. Despite the number of hypotheses of depression that have emerged through the decades—monoaminergic, neurotrophic, inflammatory, glutamatergic—to name only a few—there is still no common consensus regarding the explanation of types and symptoms of depression [3–6]. After years of research on monoaminergic transmission and the associated downstream signaling processes, Trullas and Skolnick put glutamate inhibition in the spotlight of research on depression. They observed that acute treatment of mice with MK-801 (noncompetitive N-methyl-D-aspartate receptor (NMDAR) channel blocker), 1-aminocyclopropane-carboxylic acid (ACPC) (glycine site partial agonist), or 2-amino-7-phosphonoheptanoic acid (AP-7) (competitive NMDA antagonist) decreased the immobility time in the forced swim test (FST) [7]. Their findings were supported by the observations of altered ligand binding to the NMDAR complex after the chronic administration of antidepressive agents [8]. In addition, several other NMDAR antagonists showed antidepressant-like effects in both preclinical and clinical studies [9]. Then, the discovery of the fast and sustained antidepressant activity of ketamine and subsequent studies on rapid-acting antidepressants significantly developed the concept of the neurobiology of depression [10–12]. Further research focused on ketamine, brain-derived neurotrophic factor (BDNF), and the role of neuroplasticity on the pathomechanism of depression, seemed to contradict the model of inhibition of glutamatergic signaling; this suggested the activation of glutamatergic transmission as the key mechanism behind the rapid-acting antidepressant effect [11, 13, 14]. Alongside the growing evidence supporting the role of glutamatergic signaling in the pathomechanism of depression, the altered GABAergic neurotransmission has been proven to underlie some of the symptoms of depression [15–17]. Thus, based on the aforementioned findings, the excitatory/inhibitory balance in the brain seems to be the key topic to investigate while studying potential new antidepressive agents.

The role of zinc both in neurophysiology and pathology seems tightly fixed with glutamate and GABA neurotransmission. We observe a dysregulation in zinc signaling not only in depression but also in other neuropsychological disorders, which stands in line with disruptions within the excitatory/inhibitory system. However, the causality of those interactions remains unknown.

Zinc has already been shown to block the NMDA receptor and GABA_A_ receptor (especially those containing *α* and *β* subunits) [18, 19] and appears to be an important mechanism of buffering either excessive excitation or inhibition. Therefore, it is expected that manipulating zinc intake will manifest not only as depressive-like behaviour but also as disruption on the molecular level. As already shown, the release of GABA in the hippocampus under zinc deficient conditions is decreased and the release of glutamate is increased as compared to the control [20].

In our previous studies, we reported depressive-like behaviour in GPR39-knockout mice [21, 22], decreased levels of GPR39 protein in the brain samples of suicide victims [22], and rapid-acting and long-lasting antidepressant-like effect of GPR39 agonist in CD-1 male mice [23, 24]. GPR39, first cloned in 1997 and classified as a member of the ghrelin/neurotensin family [25], was “deorphanized” in 2005 by Zhang et al., who claimed to identify obestatin as its ligand [26]. In the next few years, other researchers failed to replicate these results [27–29]. Subsequently, zinc was identified as the ligand and/or positive allosteric modulator of GPR39 [28, 30]. Interestingly, the idea of zinc being able to induce intracellular response through its specific receptor was not a new concept at that time. Hershfinkel et al. observed the zinc-induced intracellular release of Ca^2+^ in a colonocytic cell line [31] and later confirmed that the zinc-sensing receptor was GPR39 [32–34]. Due to high receptor plasticity and the lack of zinc sensitivity in fish GPR39, it is possible that another GPR39 ligand remains unrecognized [32, 35]. GPR39 is expressed in several organs, such as the pancreas, intestine, kidneys, and the brain, and is involved in processes such as insulin secretion, the formation of tight junctions, transport of ions, spermatogenesis, and bone formation [35]. Despite the growing evidence on the important role of GPR39 in the physiology of the brain, its exact function remains unclear [22, 32, 36–38].

TC-G 1008, first synthesized in 2014, is a potent GPR39 agonist-inducing response through Gαq and Gαs pathways [30, 39]. Studies are reporting the effect of GPR39 agonist on the increase in the assembly of tight junction [40], the stimulation of IL-10 production from lipopolysaccharide (LPS)-stimulated macrophages [41], decreased alcohol intake in the mouse model of binge-like ethanol drinking [42], and decreased immobility time in the FST up to 24 h following injection [23]. The mechanism behind the behavioural effects of TC-G 1008 is not clear. It is possible that GPR39 affects neurotransmitter signaling in the central nervous system (CNS), therefore we decided to investigate the role of four different receptors of glutamatergic and GABAergic systems (NMDAR, α-amino-3-hydroxy-5-methyl-4-isoxazolepropionic acid receptor (AMPAR), GABA_A_, and GABA_B_) in the antidepressant-like effect of TC-G 1008. We also aimed to evaluate the effectiveness of TC-G 1008 treatment under zinc-deficiency conditions and analyze how TC-G 1008 and zinc-deficient diet influences expression of proteins crucial in excitatory and inhibitory transmission (GABA_A_α1, GABA_A_β2, GABA_B_R1, GluN1, GluN2A, GluN2B, PSD95, KCC2).

## Materials and methods

### Animals

CD-1 male mice (20–24 g) were housed in groups of 8 per cage with free access to food and water. The 12 h light–dark cycle was maintained. Mice received a standard diet for rodents (Agropol, Poland). In the study of TC-G 1008 activity in zinc deficiency mice have received control (44 mg Zn/kg, ssniff Spezialdiäten, Germany) or low-zinc diet (≤ 5 mg Zn/kg, ssniff Spezialdiäten, Germany) for 4 (acute TC-G 1008 administration) or 6 weeks (chronic TC-G 1008 administration). All procedures were approved by the Local Institutional Animal Care and Use Committee (IACUC) in Kraków (approvals numbers: NR 160/2017 (17.05.2017), NR 61/2018 (22.02.2018), NR 62/2018 (22.02.2018)).

### Drugs

Drugs were administered intraperitoneally (*ip*) in a joint administration scheme 30 min prior to the test, except NMDA and CX614 which were injected 1 h or 15 min before the test, respectively. The time of administration was evaluated before experiments and adjusted based on our result and previous experiment performed at our department [38]. TC-G 1008 was dissolved in 1% Tween, whereas all other drugs were dissolved in saline. Control animals received 1% Tween and saline. Due to the fact that CGP 37,849, CX 614, R-baclofen, and SCH 50,911 induce an antidepressant-like response in the FST, we tested all compounds in order to determine the highest nonactive doses for the joint administration scheme (see supplementary materials for further details). Table 1 shows the list of all drugs used in this study. In our experiment we used two different doses of TC-G 1008, 10 or 15 mg/kg. Lower dose (inactive in FST) was used when TC-G 1008 was administered together with inactive doses of receptor ligands to determine potential synergistic effect between compunds. We used higher dose of TC-G 1008 (active in FST) to evaluate whether the antidepressant effect of GPR39 agonist may be blocked when administered together with NMDA, picrotoxin or NBQX (none of these compounds is active in FST).

**Table 1:** List of drugs used in the study

Name	Description	Manufacturer	Doses used	Effect of a joint administration of TC-G 1008 in the FST
TC-G 1008	GPR39 agonist	*Tocris, UK*	10 mg/kg (nonactive in the FST) 15 mg/kg (active in the FST)	**–**
NMDA	NMDAR agonist	*Sigma Aldrich, Germany*	75 mg/kg	Blocked antidepressant-like effect of TC-G 1008
CGP 37,849	NMDAR antagonist	*Sigma Aldrich, Germany*	0.65 mg/kg	No effect
NBQX	AMPAR antagonist	*Cayman Chemicals, USA*	10 mg/kg	No effect
CX 614	Positive allosteric modulator of AMPAR	*Tocris, UK*	1 mg/kg	No effect
Muscimol	GABA_A_ agonist	*Tocris, UK*	0.5 mg/kg	Trend towards decreased immobility time
Picrotoxin	GABA_A_ antagonist	*Sigma Aldrich, Germany*	1 mg/kg	Blocked antidepressant-like effect of TC-G 1008
R-Baclofen	GABA_B_ agonist	*Sigma Aldrich, Germany*	0.25 mg/kg	No effect
SCH 50,911	GABA_B_ antagonist	*Tocris, UK*	1 mg/kg	Trend toward decreased immobility time

### Forced swim test

FST was performed according to the previously described protocol [24]. Animals were placed in the glass cylinders (height 25 cm, diameter 10 cm) filled with water (23–25 °C) to the level where animals were not able to escape or support themselves with their tail. After the first 2 min of adaptation, the total immobility time was measured for the remaining 4 min of the test. Animals were considered to be immobile while floating on the water’s surface and moving only to maintain balance and keep nostrils above the water.

### Locomotor activity test

Animals were placed in plexi boxes for the total time of 6 min. Locomotor activity was measured with the Any Maze video tracking software (Stoelting, USA) as the overall distance traveled. To perform locomotor activity test we used independent groups of animals.

### Western blot

Selected brain structures were isolated 24 h after the last drug administration, immediately frozen and stored at − 80 ℃ until further testing. Tissues were homogenized in 2% SDS (sodium dodecyl sulfate) and centrifuged (1200 rpm, 20 min, 4 ℃). Next, the amount of protein in the supernatant was determined by the BCA method (Pierce ™ BCA Protein Assay Kit, Thermo Fisher Scientific, USA) using a POLARstar Omega microplate reader (BMG Labtech, Germany). The samples were diluted in 2% SDS, mixed with 2 × Laemmli buffer (Sigma Aldrich, Germany) and denatured at 95° for 5 min. Samples containing 23 or 25 μg of protein per well were electrophoretically separated (35 min, 200 V, 400 mA) on a polyacrylamide gel (10 or 12.5%) and transferred into nitrocellulose membrane by semi-dry transfer (TransBlot Turbo, Bio-Rad, USA). Next, the membranes were incubated for 1 h at room temperature in a 1% blocking reagent solution (Roche, Switzerland), and then overnight at 4 ℃ with a primary antibody in a 0.5% blocking reagent solution (see Supplementary material for the list of antibodies used). The next day, membranes were washed 3 times for 10 min in TBST buffer, incubated for one hour with secondary antibody and washed again in TBS buffer (3 times for 5 min). After incubation with the substrate for the secondary antibody-bound enzyme (Clarity ™ Western ECL Substrate, Bio-Rad, USA) the chemiluminescence was measured using a Gel Doc XR + system (Bio-Rad, USA). The band density was calculated using Image Lab 4.1 (Bio-Rad, USA).

### Statistical analysis

All data sets were checked for normal distribution (using Shapiro–Wilk normality test and Kolmogorov–Smirnov normality test) and homogeneity of variance (using Bartlett’s test and Brown-Forsythe test). All data sets were analyzed with two-way analysis of variance (ANOVA) followed by post-hoc Dunnett’s or Tukey’s tests (GraphPad Prism 7). *P* values less than 0.05 was considered as statistically significant.

## Results

### Forced swim test

In the case of all treatment schemes, TC-G 1008 administered by itself at a dose of 15 mg/kg (*ip*) decreased the immobility time, whereas TC-G 1008 at a dose of 10 mg/kg (*ip*) did not induce any changes in the immobility time. All other drugs used in this study (Table 1) administered by themselves did not affect animal behaviour in the FST.

#### The effects of joint administration of TC-G 1008 and NMDAR ligands

**Fig. 1:**
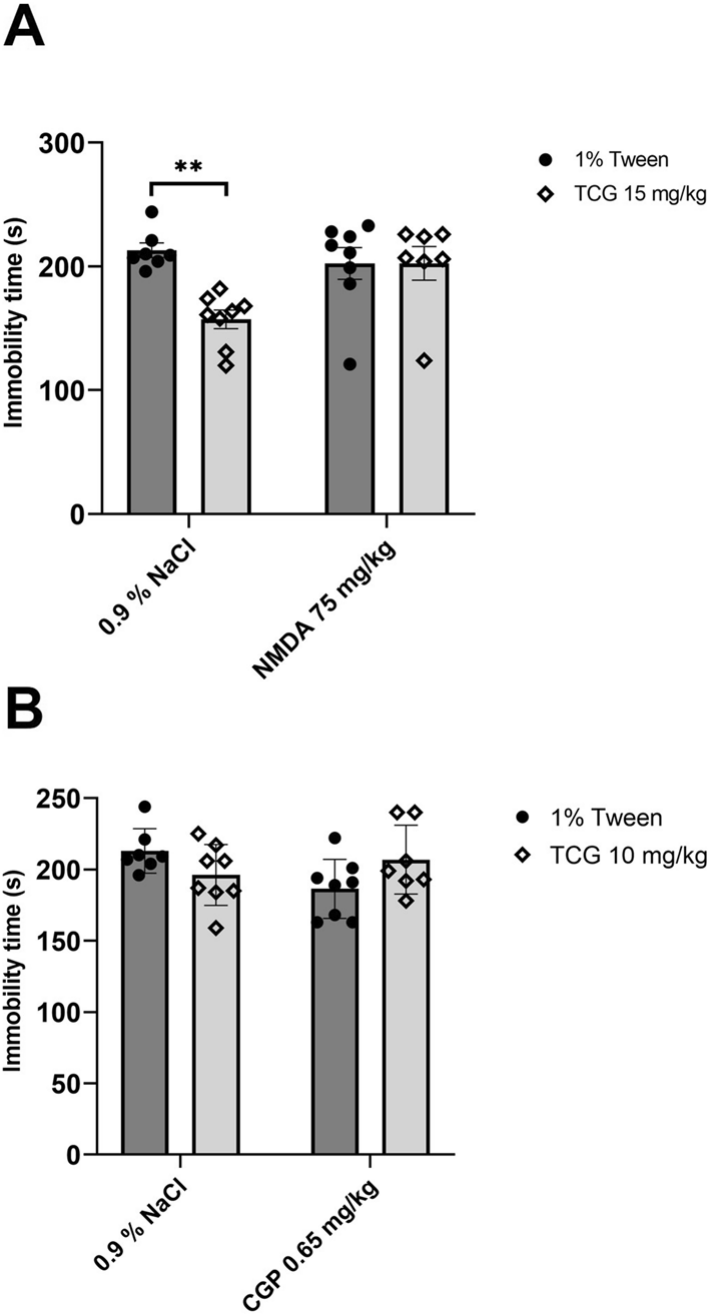
The effect of joint administration of TC-G 1008 and NMDA (*ip*) (**A**) or TC-G 1008 and CGP 37,849 (*ip*) (**B**) on immobility time in the forced swim test (FST). TC-G 1008 was administered 30 min before the test, while NMDA was administered 1 h before the test. All results are presented as mean ± SEM (n = 8). **p < 0.01 vs. 1% Tween + 0.9% NaCl (two-way analysis of variance followed by Dunnett’s multiple comparison test)

GPR39 agonist administered by itself at a dose of 15 mg/kg (*ip*) significantly decresed immobility time in the FST (*p* = 0.0026). The treatment with NMDA only (75 mg/kg) (*ip*) did not change mouse behaviour (*p* = 0.8182). TC-G 1008 (15 mg/kg) administered with NMDA (75 mg/kg) (*ip*) did not affect the immobility time when compared to the control treatment (p = 0,8334) (Fig. 1A). Two-way ANOVA showed an effect for TC-G 1008 at a dose of 15 mg/kg (*ip*) [*F*_1,26_ = 6.965; *p* = 0.0136], no effect for NMDA (*ip*) [*F*_1,26_ = 2.681; *p* = 0.1136], and an interaction between factors [*F*_1,26_ = 6.991; *p* = 0.0137].

Administration of TC-G 1008 by its own at a dose of 10 mg/kg (*ip*) or CGP 37,049 at a dose of 0.65 mg/kg (*ip*) did not affect mouse behaviour (*p* = 0.2891; *p* = 0.0512). Joint administration of TC-G 1008 (10 mg/kg) with CGP 37,849 (0.65 mg/kg) (*ip*) did not change the immobility time in the FST (Fig. 1B). Two-way ANOVA revealed no effect for TC-G 1008 at a dose of 10 mg/kg (*ip*) [*F*_1,26_ = 0.0566; *p* = 0.8138], no effect for CGP 37,849 (*ip*) [*F*_1,26_ = 1.099; *p* = 0.3042], and a significant interaction between factors [*F*_1,26_ = 6.07; *p* = 0.0207].

#### The effects of joint administration of TC-G 1008 and AMPAR ligands

**Fig. 2:**
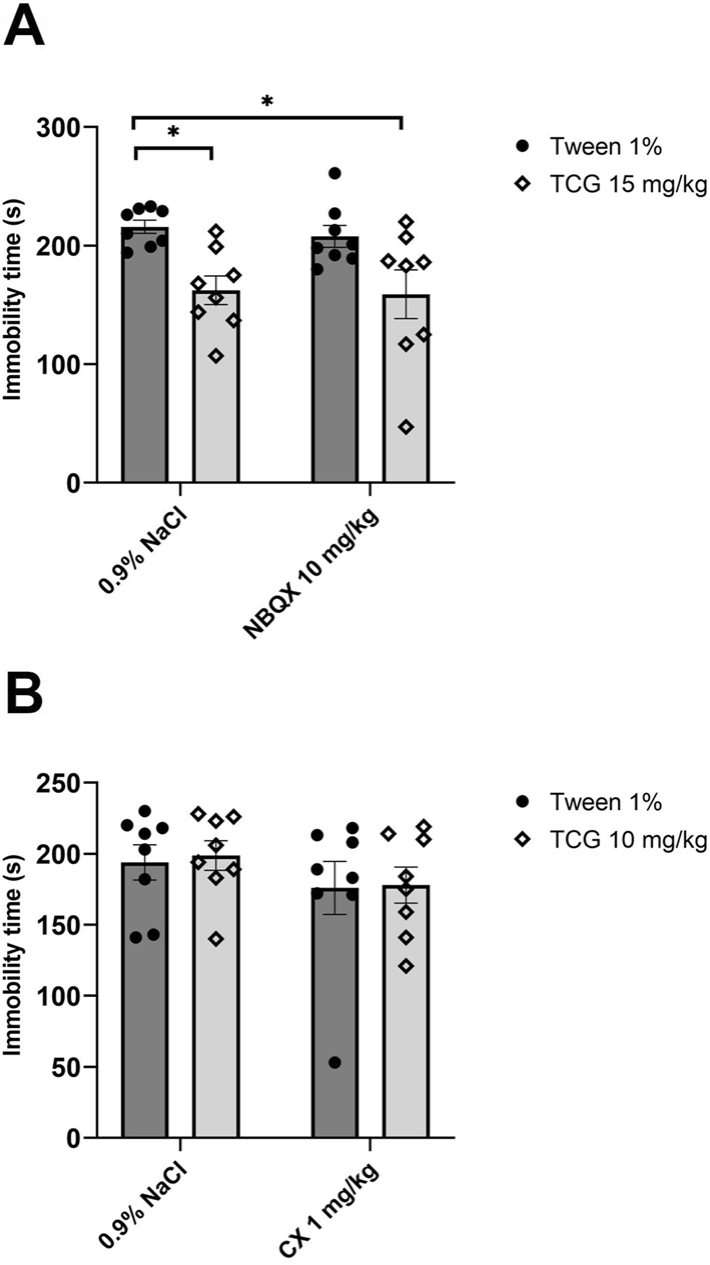
The effect of joint administration of TC-G 1008 and NBQX (*ip*) (**A**) or TC-G 1008 and CX 614 (*ip*) (**B**) on immobility time in the forced swim test (FST). All substances were administered 30 min before the test. All results are presented as mean ± SEM (n = 8). *p < 0.05 vs. 1% Tween + 0.9% NaCl (two-way analysis of variance followed by Dunnett’s multiple comparison test)

TC-G 1008 at a dose of 15 mg/kg (*ip*) significantly decreased immobility time in mice (*p* = 0.0194). NBQX (10 mg/kg) (*ip*) administered by itself did not cause any changes in the FST (*p* = 0.9461).The joint administration of NBQX (10 mg/kg) with TC-G 1008 (15 mg/kg) (*ip*) did not block the antidepressant-like effect of GPR39 agonist (*p* = 0.0127) (Fig. 2A). Two-way ANOVA showed an effect for TC-G 1008 at a dose of 15 mg/kg [*F*_1,28_ = 15.31; *p* = 0.0005], no effect for NBQX (*ip*) [*F*_1,28_ = 0.19; *p* = 0.4363], and no interaction between factors [*F*_1,28_ = 0.03489; *p* = 0.8532].

TC-G 1008 administered by itself at a dose of 10 mg/kg (*ip*) did not cause any changes in immobility time (*p* = 0.9900). CX 614 at a dose of 1 mg/kg (*ip*) also did not alter mouse behaviour (*p* = 0.6871). Joint administration of TC-G 1008 with CX 614 (*ip*) did not show any effects in the FST (*p* = 0.7546). (Fig. 2B). Two-way ANOVA revealed no effect for TC-G 1008 at a dose of 10 mg/kg (*ip*) [*F*_1,28_ = 0.05905; *p* = 0.8098], no effect for CX 614 (*ip*) [*F*_1,28_ = 1.946; *p* = 0.1740], and no interaction between factors [*F*_1,28_ = 0.009801; *p* = 0.9218].

#### The effect of joint administration of TC-G 1008 and GABA_A_ receptor ligands

**Fig. 3:**
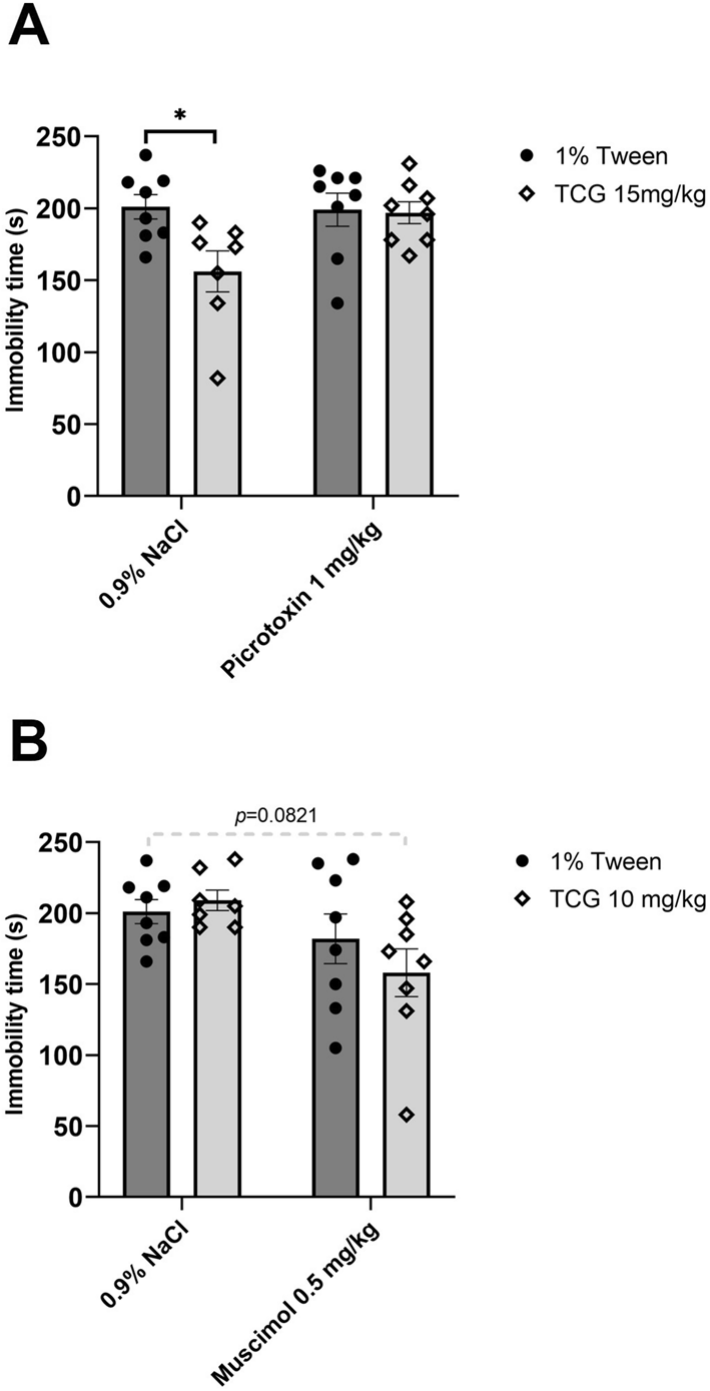
The effect of joint administration of TC-G 1008 and picrotoxin (*ip*) (**A**) or TC-G 1008 and muscimol (*ip*) (**B**) on immobility time in the forced swim test (FST). All substances were administered 30 min before the test. All results are presented as mean ± SEM (n = 8). *p < 0.05 vs. 1% Tween + 0.9% NaCl (two-way analysis of variance followed by Dunnett’s multiple comparison test)

TC-G 1008 at a dose of 15 mg/kg (*ip*) admnistered by itself decreased immobility time in the FST (*p* = 0.0174) while picrotoxin (1 mg/kg) (*ip*) did not cause any significant changes (*p* = 0.9982). The joint administration of picrotoxin (1 mg/kg) with TC-G 1008 (15 mg/kg) (*ip*) blocked the TC-G 1008-induced antidepressant-like effect in the FST when compared to control group (*p* = 0.9846) (Fig. 3A). Two-way ANOVA revealed an effect for TC-G 1008 at a dose of 15 mg/kg (*ip*) [*F*_1,27_ = 4.948; *p* = 0.0347], no effect for picrotoxin (*ip*) [*F*_1,27_ = 3.363; *p* = 0.0777], and no significant interaction between factors [*F*_1,27_ = 4.093; *p* = 0.0531].

TC-G 1008 (10 mg/kg) (*ip*) or muscimol (0.5 mg/kg) (*ip*) when administered by themselves did not change immobility time in the FST (*p* = 0.9570; *p* = 0.6289). Joint administration of TC-G 1008 (10 mg/kg) with muscimol (0.5 mg/kg) (*ip*) revealed a tendency toward decreased immobility time (*p* = 0.0821) (Fig. 3B). Two-way ANOVA showed no effect for TC-G 1008 at a dose of 10 mg/kg (*ip*) [*F*_1,27_ = 0.3342; *p* = 0.5680], an effect for muscimol (*ip*) [*F*_1,27_ = 6.522; *p* = 0.0166], and no interaction between factors [*F*_1,27_ = 1.348; *p* = 0.2559].

#### The effect of joint administration of TC-G 1008 and GABA_B_ receptor ligands

**Fig. 4:**
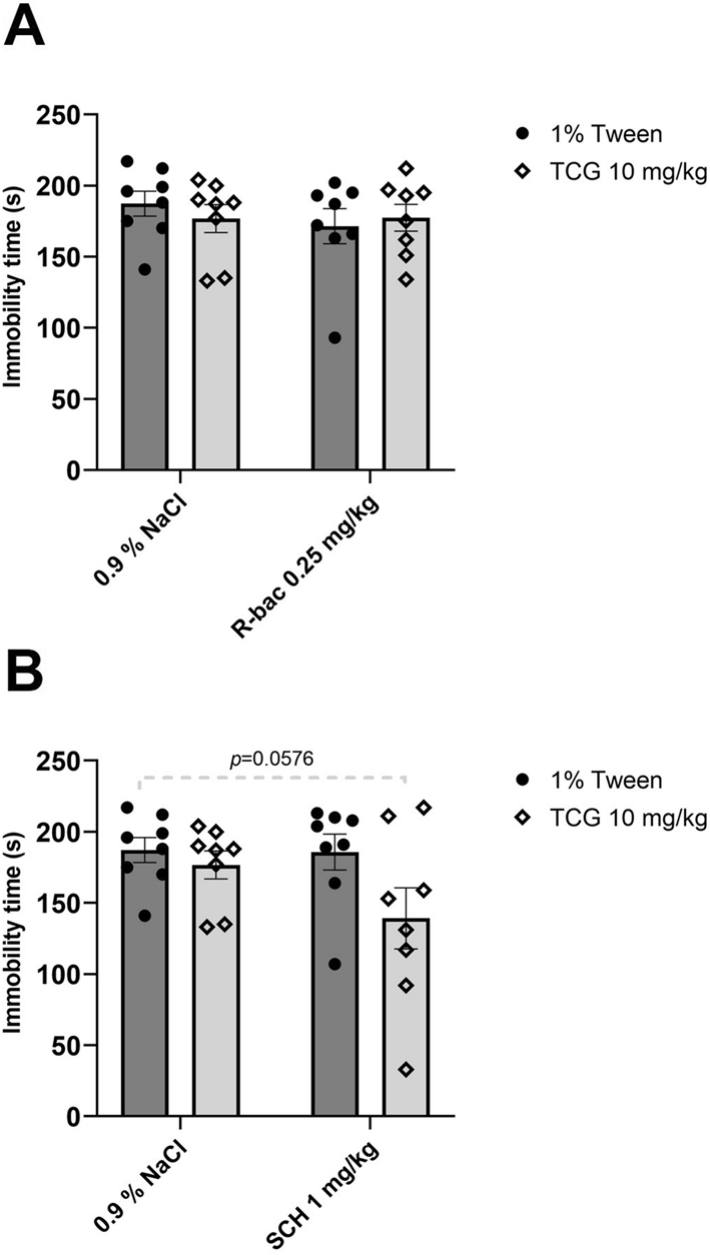
The effects of joint administration of TC-G 1008 and R-baclofen (*ip*) (**A**) or TC-G 1008 and SCH 50,911 (*ip*) (**B**) on immobility time in the forced swim test (FST). All substances were administered 30 min before the test. All results are presented as mean ± SEM (n = 8). (two-way analysis of variance followed by Dunnett’s multiple comparison test)

TC-G (10 mg/kg) (*ip*) or R-baclofen (0.25 mg/kg) (*ip*) when administered by themselves did not cause any significant changes in FST (*p* = 0.8063; *p* = 0.5583). The joint administration of TC-G 1008 with R-baclofen (*ip*) had no effect in the FST (*p* = 0.8320) (Fig. 4A). Two-way ANOVA showed no effect for TC-G 1008 in a dose of 10 mg/kg (*ip*) [*F*_1,28_ = 0.04919; *p* = 0.8261, no effect for R-baclofen (*ip*) [*F*_1,28_ = 0.5649; *p* = 0.4586] and no interaction between factors [*F*_1,28_ = 0.6613; *p* = 0.4230].

Again, administration of TC-G 1008 (10 mg/kg) (*ip*) or SCH 50,911 (1 mg/kg) (*ip*) did not change immobility time (*p* = 0.9130; *p* = 0.9996). However, joint administration of TC-G 1008 together with SCH 50,911 (*ip*) showed a tendency toward decreased immobility time in the FST (*p* = 0.0576) (Fig. 4B). Two-way ANOVA revealed no significant effect for TC-G 1008 at a dose of 10 mg/kg (*ip*) [*F*_1,28_ = 4.118; *p* = 0.0520], no effect for SCH 50,911 (*ip*) [*F*_1,28_ = 1.932; *p* = 0.1755], and no interaction between factors [*F*_1,28_ = 1.647; *p* = 0.2099].

### Locomotor activity test

**Fig. 5:**
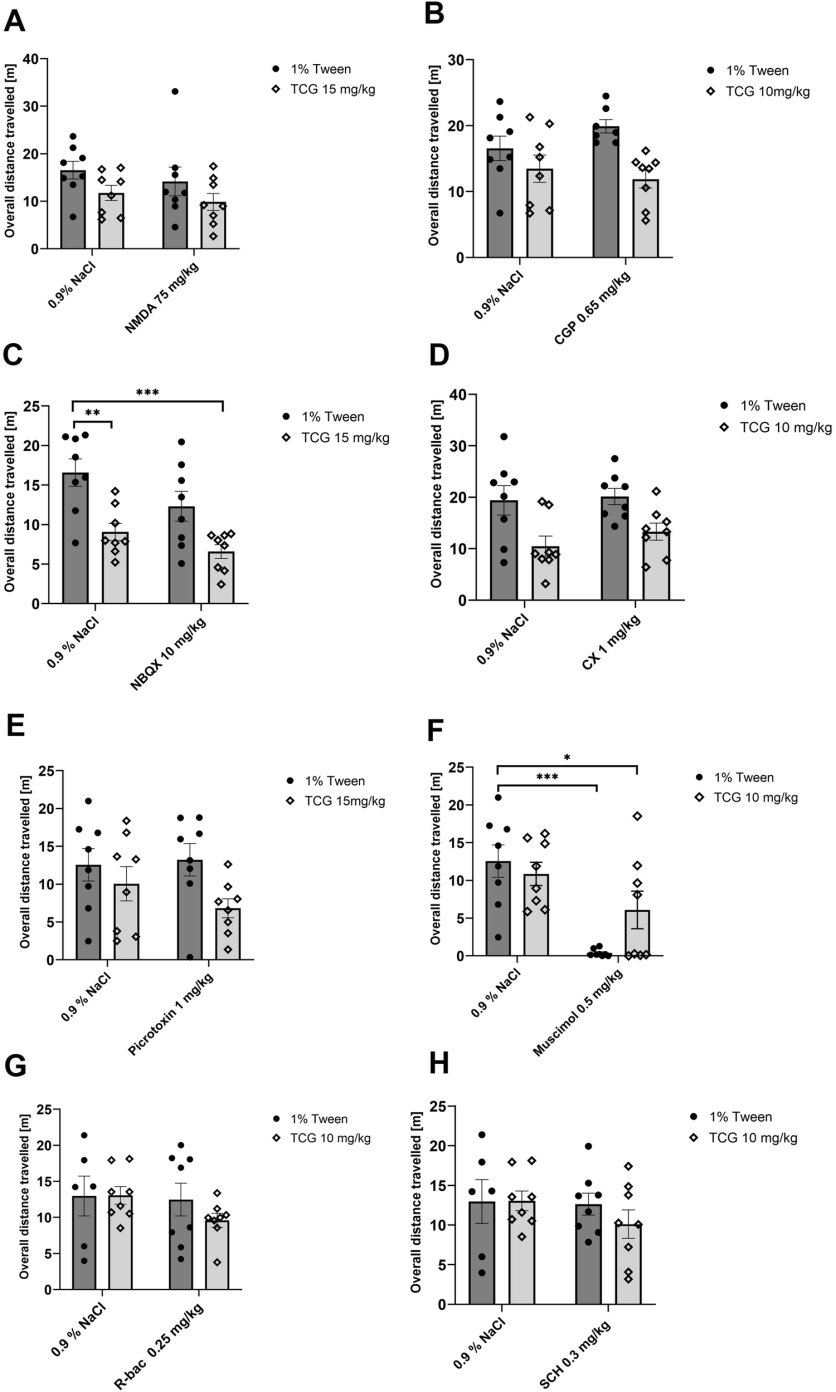
The effect of joint administration of TC-G 1008 and NMDA (*ip*) (**A**), TC-G 1008, and CGP 37,849 (*ip*) (**B**), TC-G 1008 and NBQX (*ip*) (**C**), TC-G 1008 and CX 614 (*ip*) (**D**), TC-G 1008 and picrotoxin (*ip*) (**E**), TC-G 1008 and muscimol (*ip*) (**F**), TC-G 1008 and R-baclofen (*ip*) (**G**), TC-G 1008 and SCH 50,911 (*ip*) (**H**) on locomotor activity. All substances were administered 30 min before the test, except for NMDA (administered 1 h before the test) and CX 614 (administered 15 min before the test). All results are presented as mean ± SEM (n = 8). *p < 0.05 vs. 1% Tween + 0.9% NaCl; **p < 0.01 vs. 1% Tween + 0.9% NaCl; ***p < 0.001 vs. 1% Tween + 0.9% NaCl (two-way analysis of variance followed by Dunnett’s multiple comparison test)

Locomotor activity was not increased in any of treatment schemes; however, we observed decreased mobility in a few cases (Fig. 5).

### The effect of low-zinc diet on antidepressant-like response of TC-G 1008

**Fig. 6:**
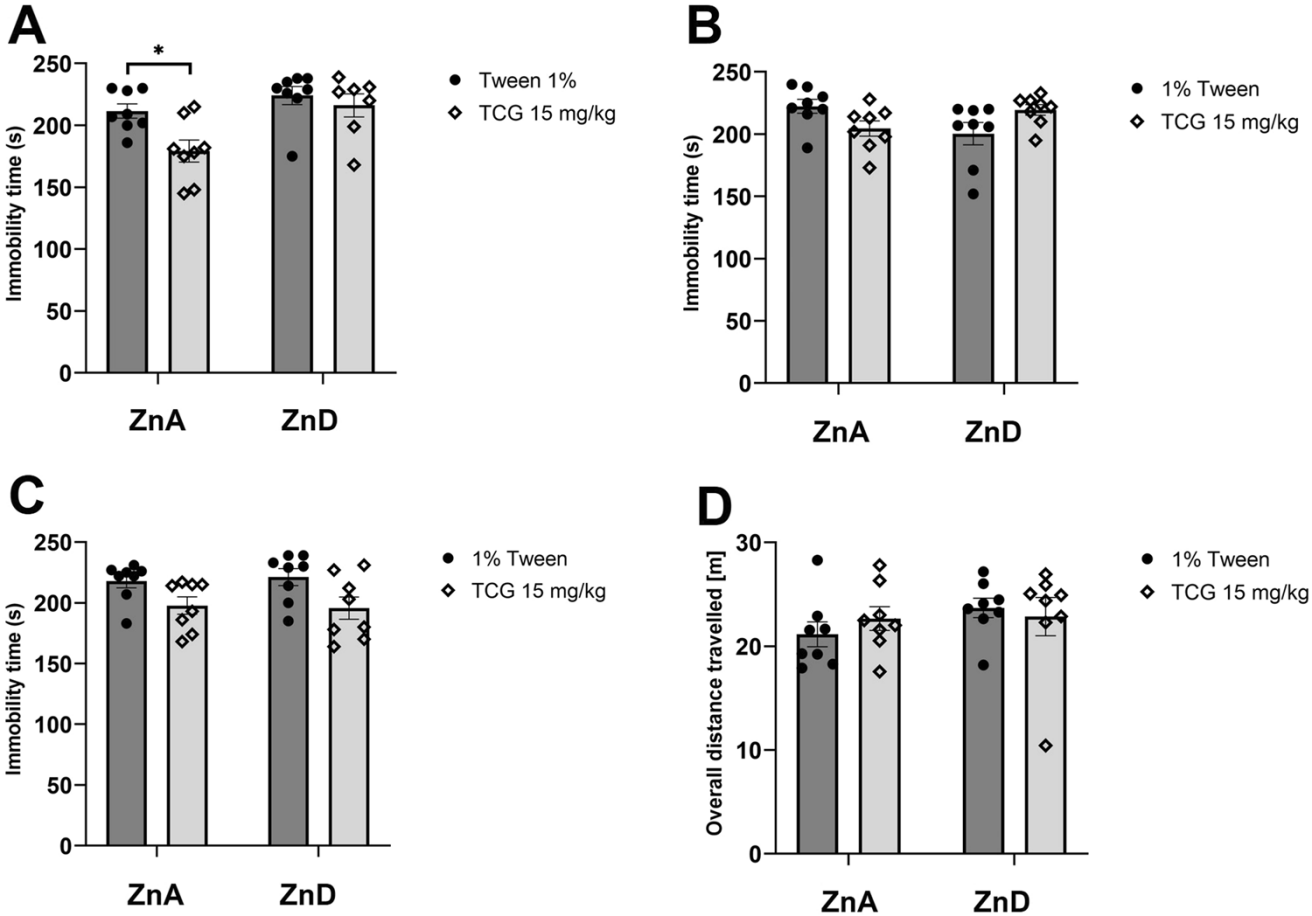
The effect of low-zinc diet on the antidepressant-like effects of TC-G 1008 in the forced swim test (FST) or locomotor activity test. 4-week low-zinc diet + acute TC-G 1008 (*ip*) treatment (FST) (**A**), 6-week low-zinc diet + acute TC-G 1008 (*ip*) treatment (FST) (**B**), 6-week low-zinc diet + chronic TC-G 1008 (*ip*) treatment (FST) (**C**), 6-week low-zinc diet + chronic TC-G 1008 (*ip*) treatment (locomotor activity) (**D**). All substances were administered 30 min before the test. All results are presented as mean ± SEM (n = 8, * p < 0.05 vs control diet + 1% Tween) (two-way analysis of variance followed by Tukey’s post-hoc test). *ZnA* control diet, *ZnD* low-zinc diet

Acute administration of TC-G 1008 (15 mg/kg) (*ip*) decreased immobility time in the FST in mice fed with 4-week control diet (*p* = 0.0309) but did not cause any changes in animals fed with 4-week low-zinc diet (Fig. 6A). Two-way ANOVA revealed the effect for TC-G 1008 (*ip*) [*F*_1,27_ = 6.544; *p* = 0.0164], effect for diet [*F*_1,27_ = 9.914; *p* = 0.0040] and no interaction between factors [*F*_1,27_ = 2.381; *p* = 0.1344].

Acute administration of TC-G 1008 (15 mg/kg) (*ip*) did not change immobility time in the FST in the groups of animals fed with 6-week zinc deficient or control diet (Fig. 6B). Two-way ANOVA showed no effect for TC-G 1008 (*ip*) [*F*_1,28_ = 0.009365; *p* = 0.9326], no effect for diet [*F*_1,28_ = 0.2937; *p* = 0.5922] and interaction between factors [*F*_1,28_ = 8.205; *p* = 0.0078].

Similarly, chronic administration of TC-G 1008 (15 mg/kg) (*ip*) did not cause any behavioural changes in the FST in the animals fed with 6-week low-zinc or control diet (Fig. 6C). Two-way ANOVA showed no effect for TC-G 1008 (*ip*) [*F*_1,28_ = 8.349; *p* = 0.0074], no effect for diet [*F*_1,28_ = 6.19; *p* = 0.9938] and no interaction between factors [*F*_1,28_ = 0.05213; *p* = 0.8211].

Chronic treatment with TC-G 1008 (15 mg/kg) (*ip*) did not cause any changes in locomotor activity of mice fed with 6-week control or experimental diet (Fig. 6D). Two-way ANOVA showed no effect for TC-G 1008 (*ip*) [*F*_1,28_ = 0.06798; *p* = 0.7962], no effect for diet [*F*_1,28_ = 1.063; *p* = 0.3114] and no interaction between factors [*F*_1,28_ = 0.7901; *p* = 0.3816].

### The effect of low-zinc diet and TC-G treatment on the levels of selected proteins in the mice brain

**Fig. 7:**
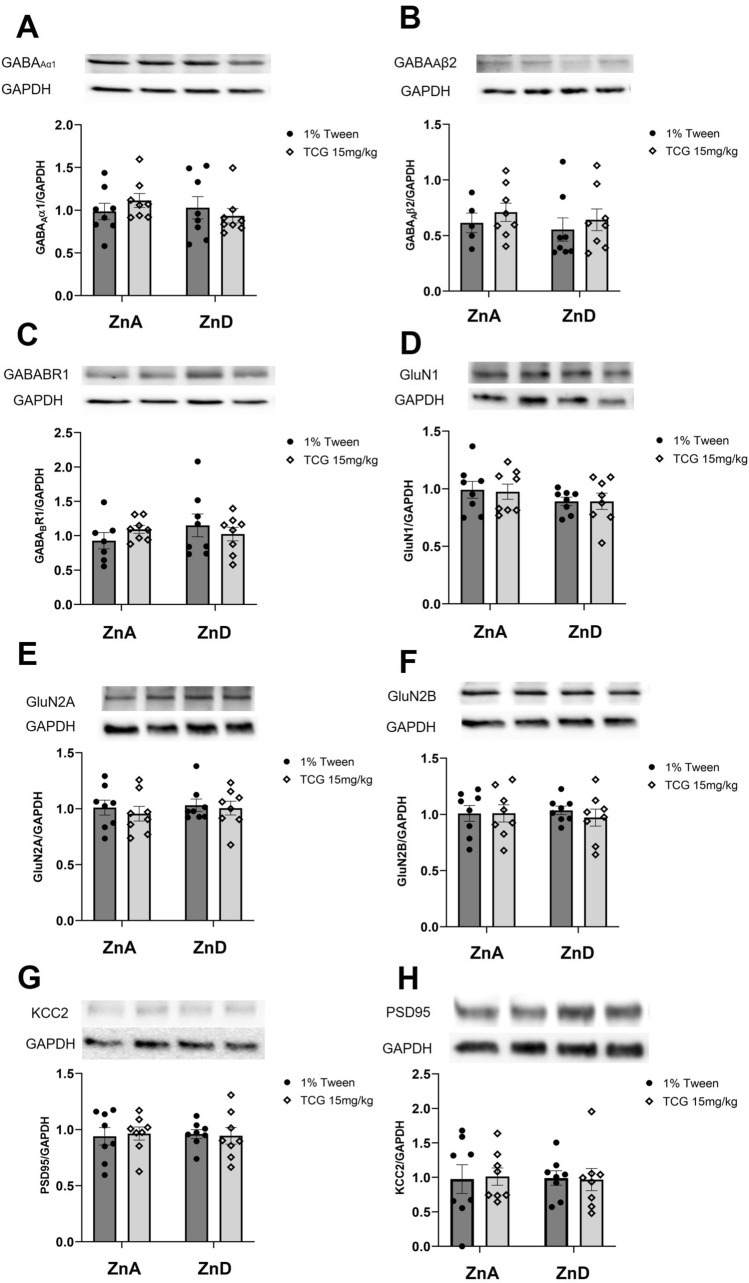
The effect of 6-week low-zinc diet and TC-G 1008 treatment on the levels of GABA_A_α1 (**A**), GABA_A_β2 (**B**), GABA_B_R1 (**C**), GluN1 (**D**), GluN2A (**E**), GluN2B (**F**), PSD95 (**G**) and KCC2 (**H**) in the hippocampus. Western Blot analysis of protein levels. All proteins were normalized do GAPDH protein levels. All results are presented as mean ± SEM (n = 7–8). (two-way analysis of variance followed by Tukey’s post-hoc test). ZnA – control diet, ZnD – low-zinc diet

**Fig. 8:**
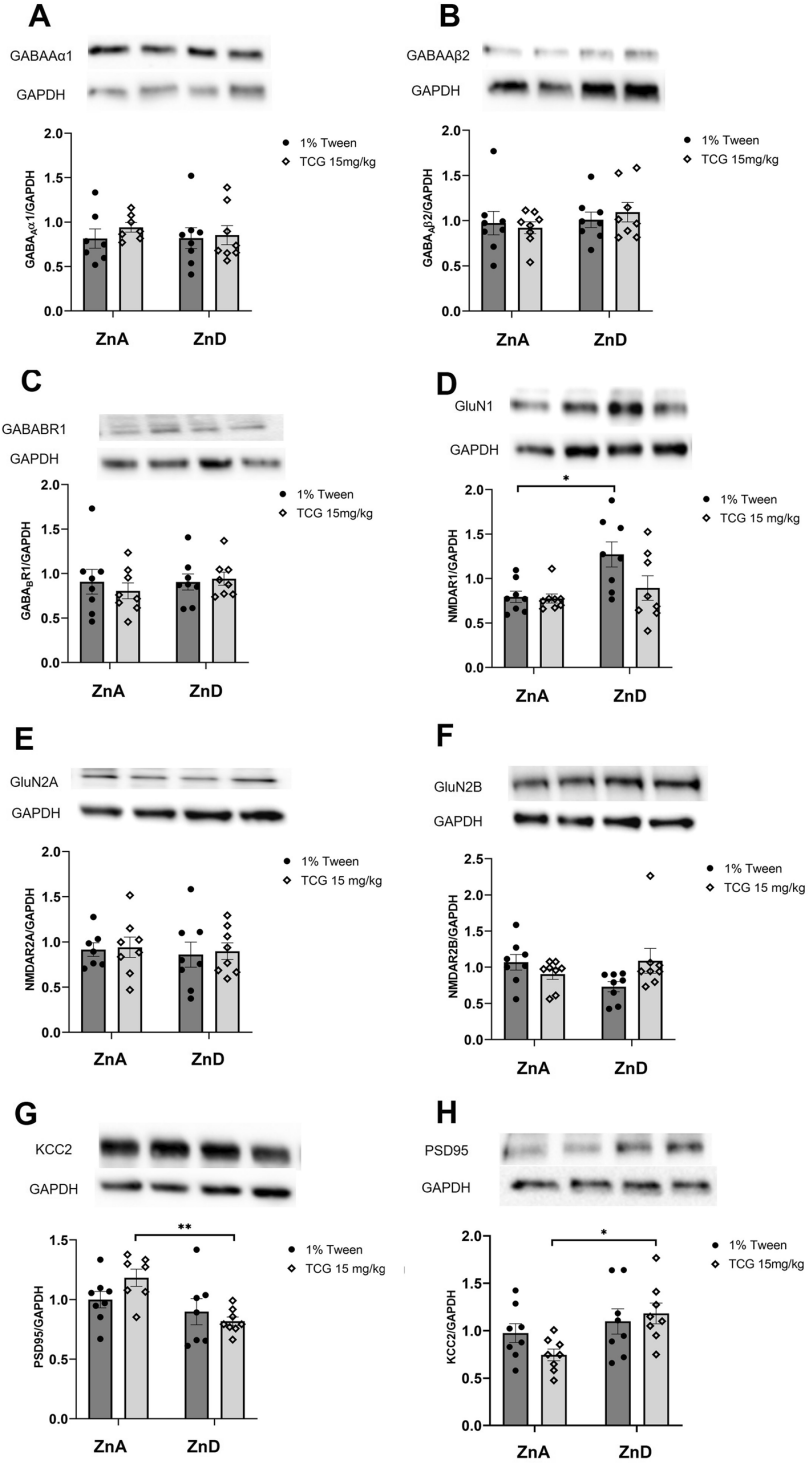
The effect of 6-week low-zinc diet and TC-G 1008 treatment on the protein levels of GABA_A_α1 (**A**), GABA_A_β2 (**B**), GABA_B_R1 (**C**), GluN1 (**D**), GluN2A (**E**), GluN2B (**F**), PSD95 (**G**), KCC2 (**H**) in the frontal cortex. Western Blot analysis of protein levels. All proteins were normalized do GAPDH protein levels. All results are presented as mean ± SEM (n = 7–8), *p < 0.05, **p < 0.01 (two-way analysis of variance followed by Tukey’s post-hoc test). ZnA – control diet, ZnD – low-zinc diet

We did not observe any changes in the protein levels of GABA_A_α1, GABA_A_β2, GABA_B_R1, GluN2A, GluN2B in both hippocampus and frontal cortex of mice treated with TC-G 1008 (15 mg/kg) (*ip*) and fed with a 6-week control or low-zinc diet (see Figs. 7 and 8).

We noticed increased level of GluN1 protein in the frontal cortex of animals which received 1% Tween solution (*ip*) and diet with reduced zinc content when compared to animals which were treated with 1% Tween (*ip*) and control diet (*p* = 0.0182). There were no chages in groups treated chronically with TC-G 1008 (15 mg/kg) (*ip*) (*p* = 0.9125).

Two-way ANOVA showed no effect for TC-G 1008 (*ip*) [*F*_1,28_ = 3.475; *p* = 0.0728], no effect for diet [*F*_1,28_ = 7.796; *p* = 0.0093] and no interaction between factors [*F*_1,28_ = 2.851; *p* = 0.1024].

### Discussion

Our previous research showed that TC-G 1008 induces fast and long-lasting antidepressant-like effects in the FST in mice [23, 24]. In this study, we evaluated the role of glutamatergic and GABAergic receptors in modifying the behavioural effect of GPR39 agonist.

The results of this study demonstrate a blocked antidepressant-like effect of TC-G 1008 when administered together with NMDA (*ip*). A number of behavioural studies have suggested that the mechanism of action of several slow-acting antidepressants is dependent on glutamatergic signalling [42]. Activation of glutamate site of NMDA receptor inhibited the effect of escitalopram, citalopram (selective serotonin reuptake inhibitor (SSRI)), and milnacipran (selective noradrenaline reuptake inhibitor (SNRI)) [43], whereas activation of the glycine site inhibited the effect of fluoxetine (SSRI), reboxetine (SNRI), and imipramine (tricyclic antidepressant (TCA)) [44]. Similarly, the antidepressant effect of zinc was blocked by the administration of NMDA [45]. However, antagonism of glutamate site enhanced the effect of escitalopram, citalopram, milnacipran, imipramine, and reboxetine [43]. Antagonism of the glycine site facilitated the effect of fluoxetine and imipramine [44]. Our results show that the antidepressant-like effect of TC-G 1008 is sensitive to NMDAR activation through the glutamate site. The role of glycine binding site in the NMDA receptor complex in the mechanism of action of GPR39 agonist is yet to be determined.

In this study, we did not observe any synergistic effect of joint (*ip*) administration of TC-G 1008 with CGP 37,849 (antagonist of the glutamate site) on the immobility time in the FST. We used the dose of CGP 37,849 that in other studies was considered as active in FST [46]. We also observed the antidepressant-like properties of this NMDA antagonist, but in a higher dose of 0.8 mg/kg. Therefore, we decided to use 0.65 mg/kg as the highest inactive dose. The lack of synergistic effect of TC-G 1008 and CGP 37,849 is consistent with our previous study [21]. MK-801 (noncompetitive NMDAR antagonist), as well as ketamine, did not require GPR39 to decrease the immobility time in the FST [21]. Although MK-801 and ketamine bind inside NMDA receptor ion channel to phencyclidine’s binding sites (which is different than the binding site for CGP 37,849), it seems that final antidepressant-like effect of NMDAR antagonists is not regulated by GPR39-mediated signaling. Furthermore, we did not prove that the effects of GPR39 agonist are directly associated with NMDA receptor function. However, we can assume that the acute administration of NMDA induces the cascade of neuronal effects which indirectly blocks the mechanism responsible for the rapid antidepressant-like response of TC-G 1008.

Prototypic rapid-acting antidepressant, ketamine, is known to exert its antidepressant effect through AMPAR-related mechanism [47, 48]. As TC-G 1008 induces rapid-acting and long-lasting effect, we decided to investigate the role of AMPA receptors in its mechanism of action. In this study, we did not find any link between the function of GPR39 and AMPA receptor.

GPR39 is known to regulate glutamate release. A previous study performed on mouse slices from dorsal cochlear nucleus GPR39 decreased the probability of glutamate release through the activation of 2-arachidonoylglycerol (2-AG) synthesis [49]. However, mouse nucleus accumbens (NAcc) slices treated with TC-G 1008 showed changes in sEPSC suggesting increased glutamate release [42]. The authors suggested that TC-G 1008 compensate ethanol-induced inhibition of neuronal activity through activation of glutamate signaling, thereby reducing alcohol intake in a mouse model of binge-like ethanol drinking [42].

Next, we investigated the role of the GABAergic system in the behavioural response following the administration of GPR39 agonist. We observed that the antidepressant-like effect of TC-G 1008 (*ip*) was blocked by picrotoxin (*ip*) but there was no significant effect when we administered TC-G 1008 together with muscimol (*ip*) (however we noted a trend toward decreased immobility time in this group). Interestingly muscimol abolished locomotor activity in a dose that normally does not induce ataxia in mice [50, 51]. We also observed significantly decreased locomotor activity following joint treatment with TC-G 1008 and muscimol (*ip*) what probably affected the outcome of the forced swim test. Chorin et al. showed that release of Zn^2+^ ions from mossy fiber terminals stimulates activity and expression of K^+^–Cl^−^ cotransporter 2 (KCC2) in pyramidal CA3 hippocampal neurons through the activation of GPR39 [33]. KCC2 acts as a major Cl^−^ extruder, maintaining low levels of intracellular Cl^−^ ions and promoting GABA_A_-/glycine receptor-mediated hyperpolarizing shift in mature neurons [52–54]. Therefore, based on our observation of the effect of joint treatment with picrotoxin, it is not surprising that the activity of GPR39 agonist seems to be dependent on GABAA receptor activity.

The role of GABA_B_ receptor in depression is not clear. In general, GABA_B_ antagonists act as antidepressant-like agents, whereas agonists are considered to be pro-depressive, but there are also exceptions to this rule [55–57]. Therefore, we decided to investigate if the joint administration of TC-G 1008 with GABA_B_ antagonist or agonist (*ip*) (all in inactive doses) may act synergistically in the FST. The joint administration of GABA_B_ antagonist resulted in a trend toward decreased immobility time. Again, although our results are on the edge of the traditional threshold for statistical significance, this observation does not exclude the possible link between the activity of these two receptors.

In the Western Blot analysis of proteins involved in glutamatergic and GABAergic transmission, we observed significant changes in GluN1, KCC2 and PSD95 protein levels in the frontal cortex. GluN1 protein level was increased in mice fed zinc-deficient diet, however there was no difference between groups that received TC-G 1008 (*ip*) (control vs zinc-deficient diet). Those results suggest that upon TC-G 1008 treatment no adaptive changes in GluN1 protein level occur as a response to zinc-deficient conditions.

The changes in protein levels of KCC2 and PSD95 were observed between groups that received different diets and TC-G 1008 (*ip*). The expression of KCC2 in frontal cortex was increased in zinc-deficient group as compared to control (both receiving TC-G 1008 (*ip*)), while the expression on PSD95 was decreased in the same group. PSD95—postsynaptic density protein 95—is a protein crucial for synaptic formation and maturation. Those processes require interaction between PSD95, NMDAR and AMPAR on postsynaptic membrane [58]. As it was reported before, this protein is highly sensitive to zinc concentration [59, 60] which is in line with our results.

KCC2 is as major outward transporter of chloride ions in neurons which activity determines the GABA_A_-inhibition. The study performed on brain slices showed GPR39-dependent upregulation of KCC2 activity in CA3 hippocampal neurons upon zinc treatment [33]. However, it is still unknown how GP39 and KCC2 interact in prefrontal cortex. From our results we can conclude, that KCC2 protein expression is affected by activation of GRP39 in zinc concentration-dependent manner.

Although the expression of GPR39 within the CNS is still a matter of discussion, it is possible that the role of this receptor is dependent on its regional and cellular localization, as well as on its physiological state. Some studies have reported that GPR39 is present in mouse hippocampus [32, 61], amygdala [61], visual cortex [61], and in nucleus accumbens core [42], as well as in human hippocampus and frontal cortex [22]. Another concern is regarding the types of neurons or glia that predominantly express GPR39. The studies performed on brain slices confirmed the presence of GPR39 (mZnR) in the pyramidal neurons of CA3 hippocampal region [32, 33] and the dorsal cochlear nucleus [49]. GPR39 is also characteristic of corticothalamic projection neurons [61]. Concerning the expression of GPR39 in glial cells, we possess limited data. Besser et al. did not detect the expression of GPR39 in astrocytes [32]. Although the number of studies related to GPR39 remains to be low, a recently released tool from the Leiden University Medical Center and TU Delft, based on the data from single-cell RNA sequencing, allows exploring the transcriptomic patterns of GPR39 in human and mouse cortex [62–64]. In the cells of the human middle temporal gyrus, mouse primary visual cortex, and anterior lateral motor area, GPR39 mRNA was found in some populations of GABAergic and glutamatergic neurons, as well as in astrocytes and oligodendrocytes. The activation of GPR39 located on pyramidal glutamate neurons may lead to a different outcome than that of the activation of receptors located on interneurons. One of the theories behind the rapid antidepressant effect of ketamine assumes that it targets interneuronal and extrasynaptic NMDA receptors, leading to the disinhibition of pyramidal neurons [11]. Activation of GPR39 might induce a similar effect which seems to be associated with the activation of interneuronal GABAA receptors rather than the inhibition of NMDA receptors. Activation of interneuronal GPR39 following the stimulation of presynaptic GABAA-ergic signaling and disinhibition of pyramidal neurons might explain the TC-G 1008-induced bias toward excitation observed by Carlson et al. in NAcc slices [42]. Although interesting, this idea can only be speculated and is a good starting point for further research.

## Conclusion

The results of this study demonstrate that the mechanism behind the antidepressant-like effect of TC-G 1008 is dependent on GABAergic and glutamatergic signaling and support the idea that GPR39 is involved in the regulation of excitatory/inhibitory balance in the brain. In our opinion, the discovery of novel modulators of the GPR39 should be considered an interesting and promising goal in the development of new psychiatric drugs.

## Supplementary Information

Below is the link to the electronic supplementary material.

Supplementary file1 (DOCX 5537 KB)

Supplementary file2 (DOCX 5343 KB)

## Data Availability

The datasets generated during and/or analyzed during the current study are available from the corresponding author on reasonable request. All immunoblots generated during the study are also included in Supplementary Materials.

## Abbreviations

ACPC: 1-Aminocyclopropane-carboxylic acid
AMPA: α-Amino-3-hydroxy-5-methyl-4-isoxazolepropionic acid
AP-7: 2-Amino-7-phosphonoheptanoic acid
BDNF: Brain-derived neurotropic factor
CNS: Central nervous system
FST: Forced swim test
GABA: Gamma-aminobutyric acid
*ip*: Intraperitoneally
KCC2: K^+^–Cl^−^ cotransporter 2
NMDA: *N*-Methyl-d-aspartate
PSD95: Postsynaptic density protein 95
RAAD: Rapid-acting antidepressant
SNRI: Selective noradrenaline reuptake inhibitor
SSRI: Selective serotonin reuptake inhibitor
TCA: Tricyclic antidepressant
