# Supplementary material for: The role of GPR39 zinc receptor in the modulation of glutamatergic and GABAergic transmission
Source: Pharmacol Rep. 2023 Mar 30;75(3):609–22. doi: 10.1007/s43440-023-00478-0 (PMC10227137; doi:10.1007/s43440-023-00478-0)
Supplement: Supplementary file 2 — Supplementary file2 (DOCX 5343 KB) [file 43440_2023_478_MOESM2_ESM.docx]

**The role of GPR39 zinc receptor in modulation of glutamatergic and GABAergic transmission**

**Supplementary materials**

In all immunoblots samples were loaded as follows:


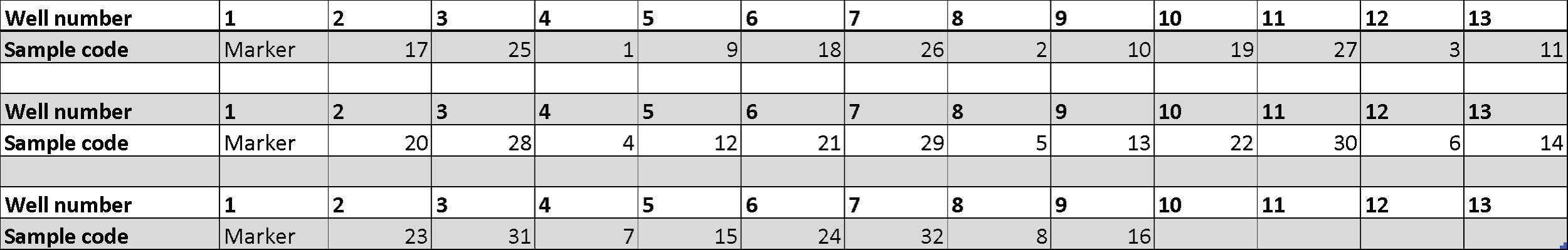


| **Group** | **Sample code** |
| --- | --- |
| ZnA + 1%TWEEN | 17 |
| ZnA + 1%TWEEN | 18 |
| ZnA + 1%TWEEN | 19 |
| ZnA + 1%TWEEN | 20 |
| ZnA + 1%TWEEN | 21 |
| ZnA + 1%TWEEN | 22 |
| ZnA + 1%TWEEN | 23 |
| ZnA + 1%TWEEN | 24 |
| ZnA + TC-G 1008 (15mg/kg) | 25 |
| ZnA + TC-G 1008 (15mg/kg) | 26 |
| ZnA + TC-G 1008 (15mg/kg) | 27 |
| ZnA + TC-G 1008 (15mg/kg) | 28 |
| ZnA + TC-G 1008 (15mg/kg) | 29 |
| ZnA + TC-G 1008 (15mg/kg) | 30 |
| ZnA + TC-G 1008 (15mg/kg) | 31 |
| ZnA + TC-G 1008 (15mg/kg) | 32 |
| ZnD + 1%TWEEN | 1 |
| ZnD + 1%TWEEN | 2 |
| ZnD + 1%TWEEN | 3 |
| ZnD + 1%TWEEN | 4 |
| ZnD + 1%TWEEN | 5 |
| ZnD + 1%TWEEN | 6 |
| ZnD + 1%TWEEN | 7 |
| ZnD + 1%TWEEN | 8 |
| ZnD + TC-G 1008 (15mg/kg) | 9 |
| ZnD + TC-G 1008 (15mg/kg) | 10 |
| ZnD + TC-G 1008 (15mg/kg) | 11 |
| ZnD + TC-G 1008 (15mg/kg) | 12 |
| ZnD + TC-G 1008 (15mg/kg) | 13 |
| ZnD + TC-G 1008 (15mg/kg) | 14 |
| ZnD + TC-G 1008 (15mg/kg) | 15 |
| ZnD + TC-G 1008 (15mg/kg) | 16 |

Table 1. Antibodies used in Western Blot analysis

| Protein | Primary antibody | Secondary antibody |
| --- | --- | --- |
| KCC2 | Anti-KCC2 (ab49917)  Rabbit polyclonal  (1:1000, Abcam, UK) | Anti-mouse IgG-POD/ant-  rabbit IgG-POD  (1:12500, Roche, USA) |
| GABAA beta2 | Anti-GABAAR β2 (ab8340)  Rabbit polyclonal  (1:1000, Abcam, UK) |  |
| GABAA alfa1 | Anti-GABAAR α1 (ab33299)  Rabbit polyclonal  (1:10000, Abcam, UK) |  |
| GluN2A | Anti-NMDAR2A (ab124913),  Rabbit polyclonal  (1:1000, Abcam, UK) |  |
| GluN2B | Anti-NMDAR2B (ab65783)  Rabbit polyclonal  (1:1000, Abcam, UK) |  |
| PSD95 | Anti-PSD95 (ab13552)  Mouse monoclonal  (1:1000, Abcam, UK) |  |
| GluN1 | Anti-NMDAR1 (ab109182)  Rabbit polyclonal  (1:1000, Abcam, UK) |  |
| GABABR1 | Anti-GABABR1 (ab55051)  Mouse monoclonal  (1:100, Abcam, UK) |  |
| GAPDH | Anti-GAPDH (sc-32233)  Mouse monoclonal  (1:200, Santa Cruz Biotechnology) | Mouse IgG kappa binding protein BP-HRP (sc-516102)  (1:1000, Santa Cruz  Biotechnology) |

Figure 1.

Structure: FCX Protein: KCC2 Reference: GAPDH

| Gel number | KCC2 | GAPDH |
| --- | --- | --- |
| Gel 1 series I  Gel 2 series I  Gel 3 series I | 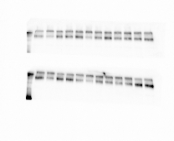  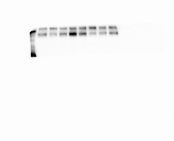 | 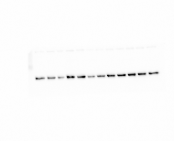  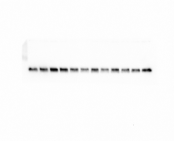  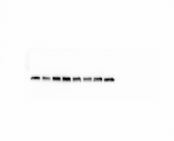 |
| Gel 1 series II  Gel 2 series II  Gel 3 series II | 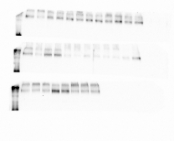 | 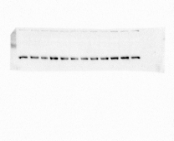  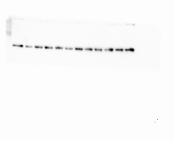  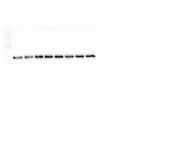 |
| Gel 1 series III  Gel 2 series III  Gel 3 series III | 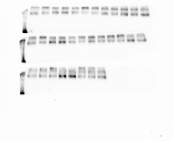 | 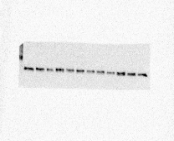  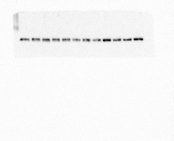  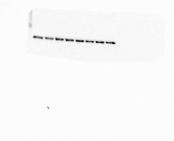 |

Figure 1.

Raw blot images for KCC2 protein and GAPDH protein in the frontal cortex. All samples contained 23 or 25 μg of protein per well. Analysis was conducted in ImageLab 4.1 (Bio-Rad, USA).

Figure 2.

Structure: FCX Protein: GABAA beta2 Reference: GAPDH

| Gel number | GABAA beta2 | GAPDH |
| --- | --- | --- |
| Gel 1 series I  Gel 2 series I  Gel 3 series I | 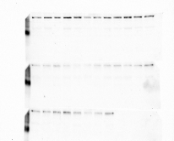 | 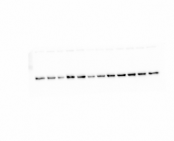  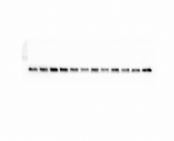  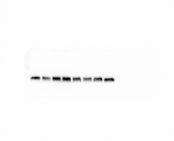 |
| Gel 1 series II  Gel 2 series II  Gel 3 series II | 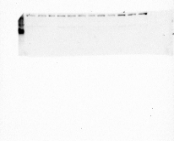  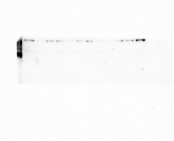  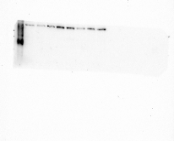 | 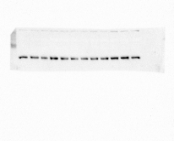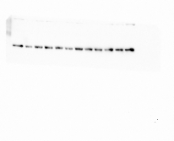  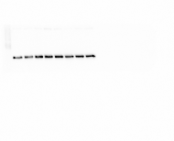 |
| Gel 1 series III  Gel 2 series III  Gel 3 series III | 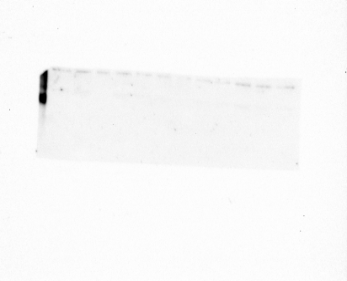  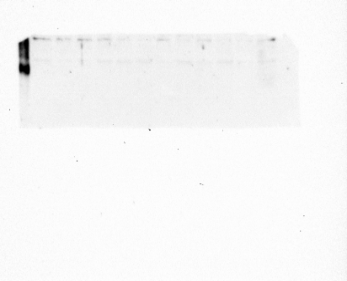  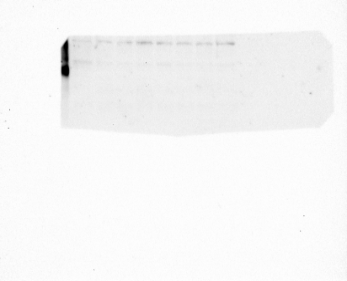 | 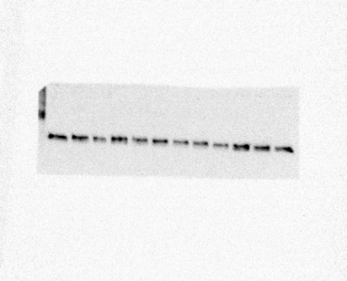  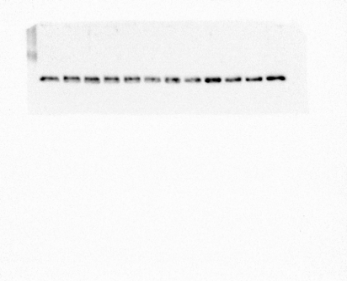  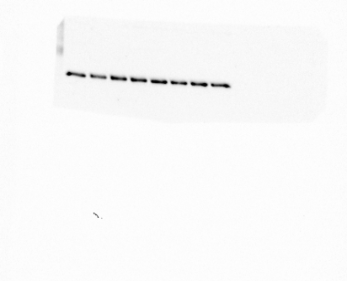 |

Figure 2.

Raw blot images for GABAA beta2 protein and GAPDH protein in the frontal cortex. All samples contained 23 or 25 μg of protein per well. Analysis was conducted in ImageLab 4.1 (Bio-Rad, USA).

Figure 3.

Structure: FCX Protein: GABAA alfa1 Reference: GAPDH

| Gel number | GABAA alfa1 | GAPDH |
| --- | --- | --- |
| Gel 1 series I  Gel 2 series I  Gel 3 series I | 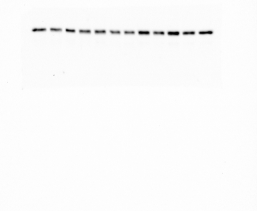  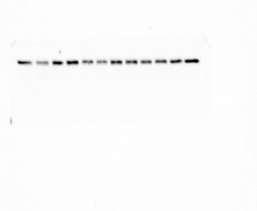  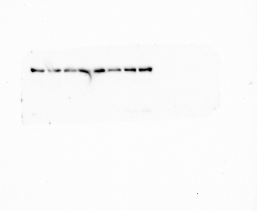 | 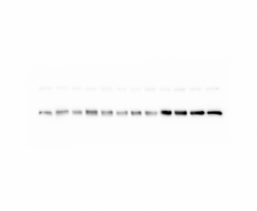  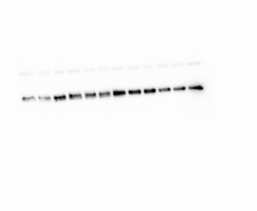  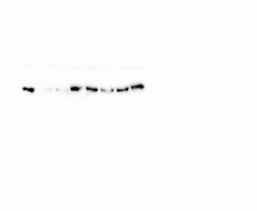 |
| Gel 1 series II  Gel 2 series II  Gel 3 series II | 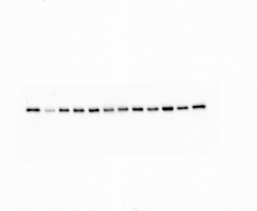  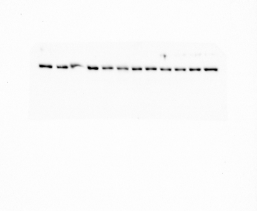  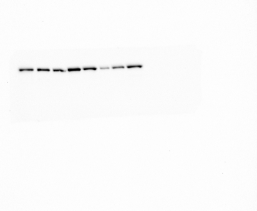 | 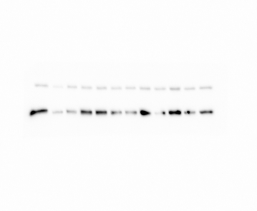  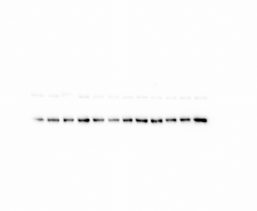  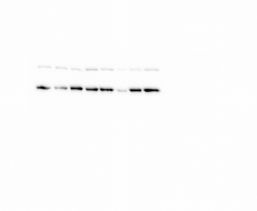 |
| Gel 1 series III  Gel 2 series III  Gel 3 series III | 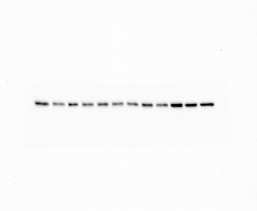  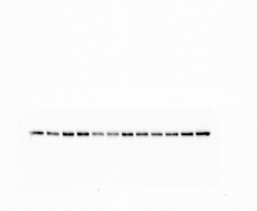  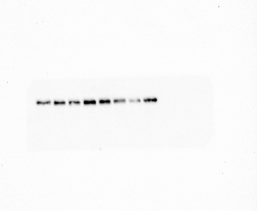 | 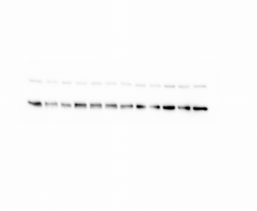  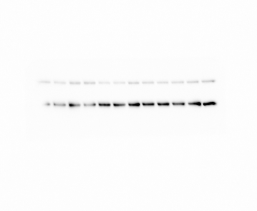  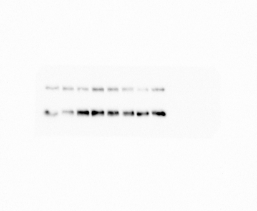 |

Figure 3.

Raw blot images for GABAA alfa1 protein and GAPDH protein in the frontal cortex. All samples contained 23 or 25 μg of protein per well. Analysis was conducted in ImageLab 4.1 (Bio-Rad, USA).

Figure 4.

Structure: FCX Protein: GluN2A Reference: GAPDH

| Gel number | GluN2A | GAPDH |
| --- | --- | --- |
| Gel 1 series I  Gel 2 series I  Gel 3 series I | 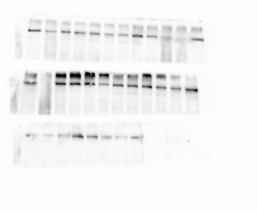 | 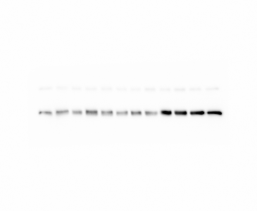  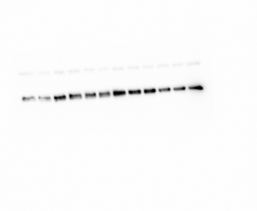  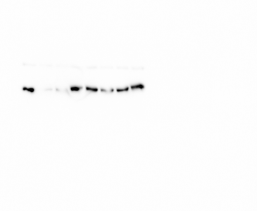 |
| Gel 1 series II  Gel 2 series II  Gel 3 series II | 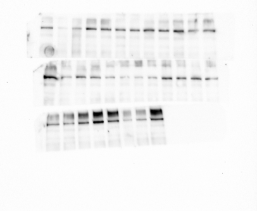 | 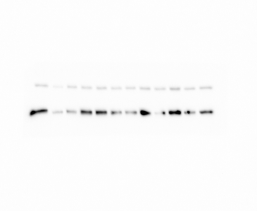  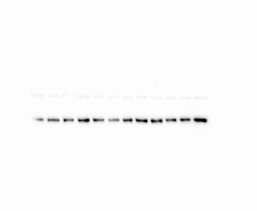  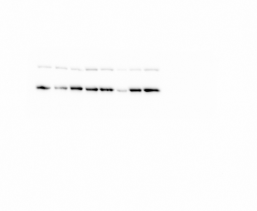 |
| Gel 1 series III  Gel 2 series III  Gel 3 series III | 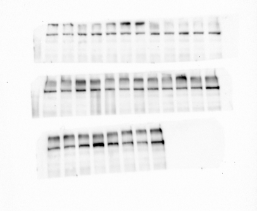 | 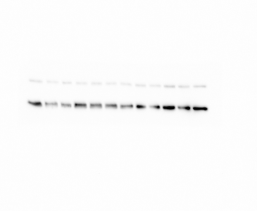  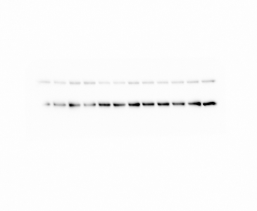  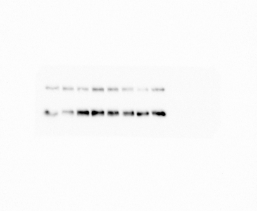 |

Figure 4.

Raw blot images for GluN2A protein and GAPDH protein in the frontal cortex. All samples contained 23 or 25 μg of protein per well. Analysis was conducted in ImageLab 4.1 (Bio-Rad, USA).

Figure 5.

Structure: FCX Protein: GluN2B Reference: GAPDH

| Gel number | GluN2B | GAPDH |
| --- | --- | --- |
| Gel 1 series I  Gel 2 series I  Gel 3 series I | 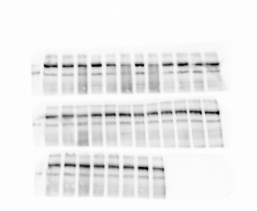 | 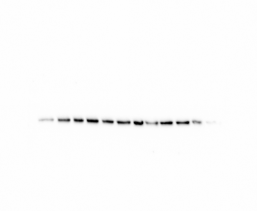  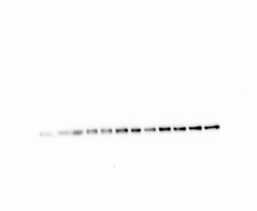  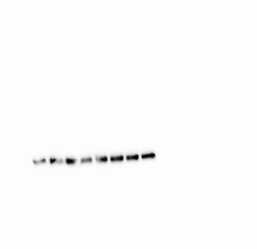 |
| Gel 1 series II  Gel 2 series II  Gel 3 series II | 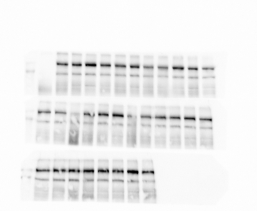 | 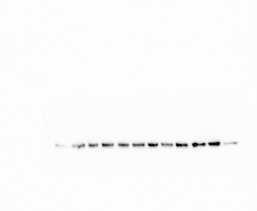  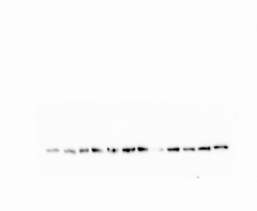  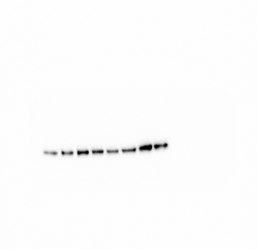 |
| Gel 1 series III  Gel 2 series III  Gel 3 series III | 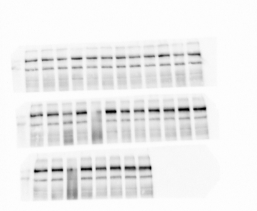 | 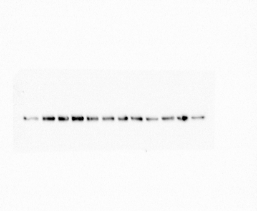  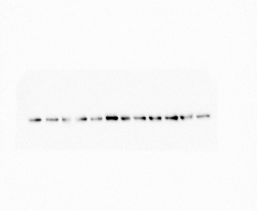  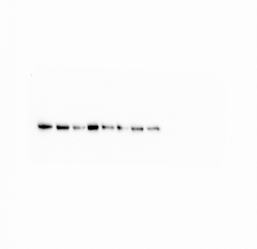 |

Figure 5.

Raw blot images for GluN2B protein and GAPDH protein in the frontal cortex. All samples contained 23 or 25 μg of protein per well. Analysis was conducted in ImageLab 4.1 (Bio-Rad, USA).

Figure 6.

Structure: FCX Protein: PSD95 Reference: GAPDH

| Gel number | PSD95 | GAPDH |
| --- | --- | --- |
| Gel 1 series I  Gel 2 series I  Gel 3 series I | 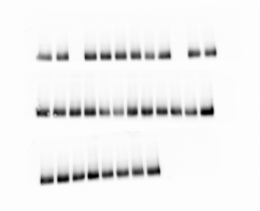 | 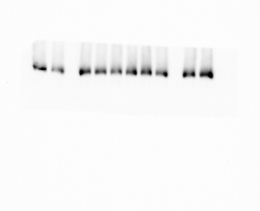  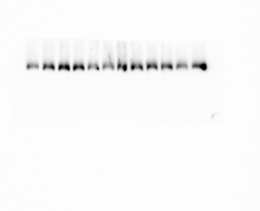  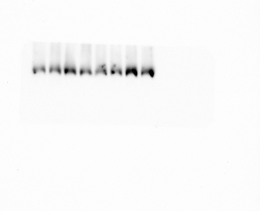 |
| Gel 1 series II  Gel 2 series II  Gel 3 series II | 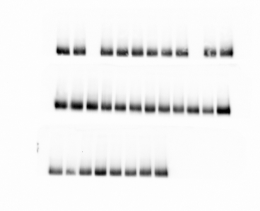 | 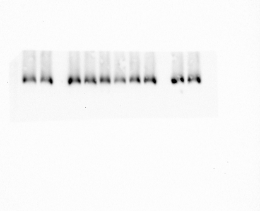  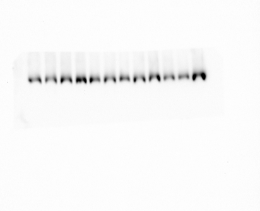  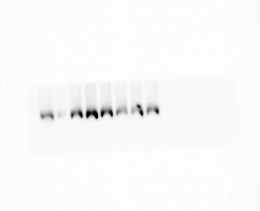 |
| Gel 1 series III  Gel 2 series III  Gel 3 series III | 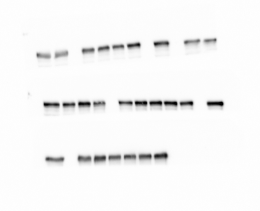 | 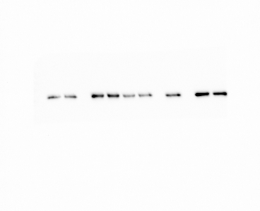  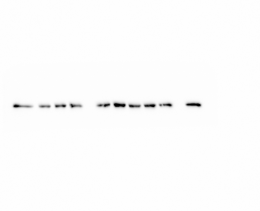  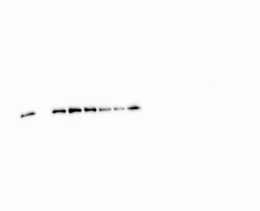 |

Figure 6.

Raw blot images for PSD95 protein and GAPDH protein in the frontal cortex. All samples contained 23 or 25 μg of protein per well. Analysis was conducted in ImageLab 4.1 (Bio-Rad, USA).

Figure 7.

Structure: FCX Protein: GluN1 Reference: GAPDH

| Gel number | GluN1 | GAPDH |
| --- | --- | --- |
| Gel 1 series I  Gel 2 series I  Gel 3 series I | 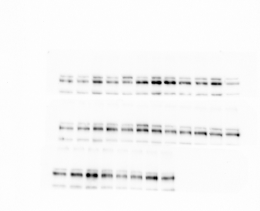 | 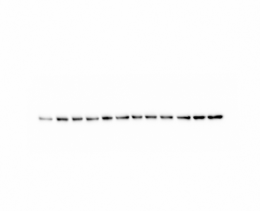  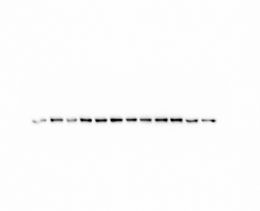  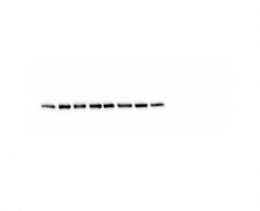 |
| Gel 1 series II  Gel 2 series II  Gel 3 series II | 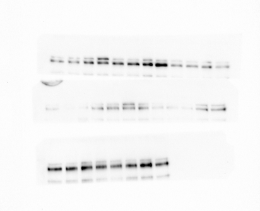 | 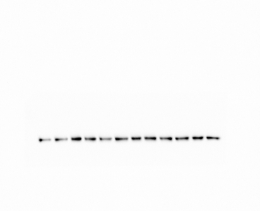  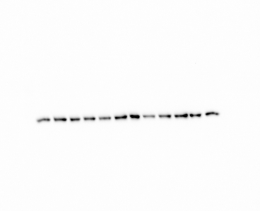  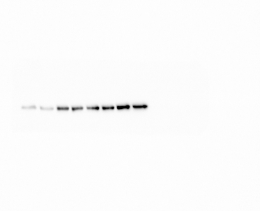 |
| Gel 1 series III  Gel 2 series III  Gel 3 series III | 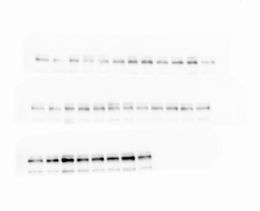 | 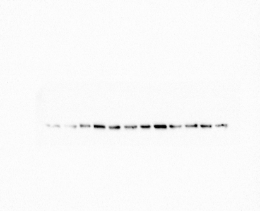  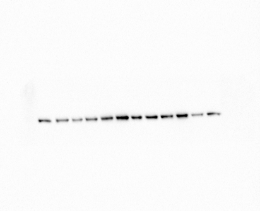  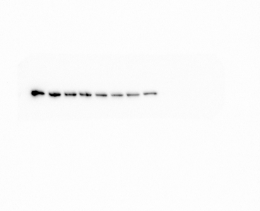 |

Figure 7.

Raw blot images for GluN1 protein and GAPDH protein in the frontal cortex. All samples contained 23 or 25 μg of protein per well. Analysis was conducted in ImageLab 4.1 (Bio-Rad, USA).

Figure 8.

Structure: FCX Protein: GABABR1 Reference: GAPDH

| Gel number | GABABR1 | GAPDH |
| --- | --- | --- |
| Gel 1 series I  Gel 2 series I  Gel 3 series I | 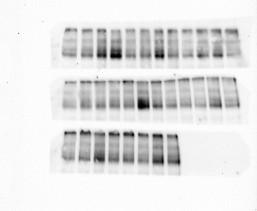 | 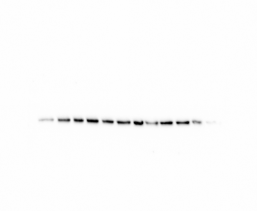  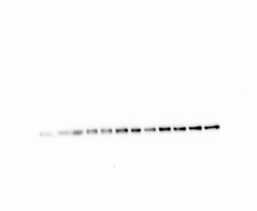  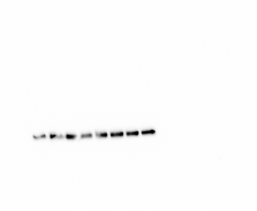 |
| Gel 1 series II  Gel 2 series II  Gel 3 series II |  |  |
| Gel 1 series III  Gel 2 series III  Gel 3 series III |  |  |

Figure 8.

Raw blot images for GABABR1 protein and GAPDH protein in the frontal cortex. All samples contained 23 or 25 μg of protein per well. Analysis was conducted in ImageLab 4.1 (Bio-Rad, USA).

Figure 9.

Structure: HP Protein: GABAA alpha1 Reference: GAPDH

| Gel number | GABAA alpha1 | GAPDH |
| --- | --- | --- |
| Gel 1 series I  Gel 2 series I  Gel 3 series I |  |  |
| Gel 1 series II  Gel 2 series II  Gel 3 series II |  |  |
| Gel 1 series III  Gel 2 series III  Gel 3 series III |  |  |

Figure 9.

Raw blot images for GABAA alpha1 protein and GAPDH protein in the hippocampus. All samples contained 23 or 25 μg of protein per well. Analysis was conducted in ImageLab 4.1 (Bio-Rad, USA).

Figure 10.

Structure: HP Protein: GluN2A Reference: GAPDH

| Gel number | GluN2A | GAPDH |
| --- | --- | --- |
| Gel 1 series I  Gel 2 series I  Gel 3 series I |  |  |
| Gel 1 series II  Gel 2 series II  Gel 3 series II |  |  |
| Gel 1 series III  Gel 3 series III  Gel 2 series III |  |  |

Figure 10.

Raw blot images for GluN2A protein and GAPDH protein in the hippocampus. All samples contained 23 or 25 μg of protein per well. Analysis was conducted in ImageLab 4.1 (Bio-Rad, USA).

Figure 11.

Structure: HP Protein: GluN2B Reference: GAPDH

| Gel number | GluN2B | GAPDH |
| --- | --- | --- |
| Gel 1 series I  Gel 2 series I  Gel 3 series I |  |  |
| Gel 1 series II  Gel 2 series II  Gel 3 series II |  |  |
| Gel 1 series III  Gel 2 series III  Gel 3 series III |  |  |

Figure 11.

Raw blot images for GluN2B protein and GAPDH protein in the hippocampus. All samples contained 23 or 25 μg of protein per well. Analysis was conducted in ImageLab 4.1 (Bio-Rad, USA).

Figure 12.

Structure: HP Protein: PSD95 Reference: GAPDH

| Gel number | PSD95 | GAPDH |
| --- | --- | --- |
| Gel 1 series I  Gel 2 series I  Gel 3 series I |  |  |
| Gel 1 series II  Gel 2 series II  Gel 3 series II |  |  |
| Gel 1 series III  Gel 2 series III  Gel 3 series III |  | Due to the difficulty with second membrane, we only included results from 1. and 3. membrane. |

Figure 12.

Raw blot images for PSD95 protein and GAPDH protein in the hippocampus. All samples contained 23 or 25 μg of protein per well. Analysis was conducted in ImageLab 4.1 (Bio-Rad, USA).

Figure 13.

Structure: HP Protein: GABAA beta2 Reference: GAPDH

| Gel number | GABAA beta2 | GAPDH |
| --- | --- | --- |
| Gel 1 series I  Gel 2 series I  Gel 3 series I |  |  |
| Gel 1 series II  Gel 2 series II  Gel 3 series II |  |  |
| Gel 1 series III  Gel 2 series III  Gel 3 series III |  |  |

Figure 13.

Raw blot images for GABAA beta2 protein and GAPDH protein in the hippocampus. All samples contained 23 or 25 μg of protein per well. Analysis was conducted in ImageLab 4.1 (Bio-Rad, USA).

Figure 14.

Structure: HP Protein: GluN1 Reference: GAPDH

| Gel number | GluN1 | GAPDH |
| --- | --- | --- |
| Gel 1 series I  Gel 2 series I  Gel 3 series I |  |  |
| Gel 1 series II  Gel 2 series II  Gel 3 series II |  |  |
| Gel 1 series III  Gel 2 series III  Gel 3 series III |  |  |

Figure 14.

Raw blot images for GluN1 protein and GAPDH protein in the hippocampus. All samples contained 23 or 25 μg of protein per well. Analysis was conducted in ImageLab 4.1 (Bio-Rad, USA).

Figure 15.

Structure: HP Protein: KCC2 Reference: GAPDH

| Gel number | KCC2 | GAPDH |
| --- | --- | --- |
| Gel 2 series I  Gel 1 series I  Gel 3 series I |  |  |
| Gel 1 series II  Gel 2 series II  Gel 3 series II |  |  |
| Gel 1 series III  Gel 2 series III  Gel 3 series III |  |  |

Figure 15.

Raw blot images for KCC2 protein and GAPDH protein in the hippocampus. All samples contained 23 or 25 μg of protein per well. Analysis was conducted in ImageLab 4.1 (Bio-Rad, USA).

Figure 16.

Structure: HP Protein: GABAB R1 Reference: GAPDH

| Gel number | GABAB R1 | GAPDH |
| --- | --- | --- |
| Gel 1 series I  Gel 2 series I  Gel 3 series I |  |  |
| Gel 1 series II  Gel 2 series II  Gel 3 series II |  |  |
| Gel 1 series III  Gel 2 series III  Gel 3 series III |  |  |

Figure 16.

Raw blot images for GABAB R1 protein and GAPDH protein in the hippocampus. All samples contained 23 or 25 μg of protein per well. Analysis was conducted in ImageLab 4.1 (Bio-Rad, USA).

Table 2. Locomotor activity results for the effect of joint administration of TC-G 1008 and NMDA (*ip*). Locomotor activity was measured with the Any Maze video tracking software (Stoelting, USA) as the overall distance travelled.

Table 3. Locomotor activity results for the effect of joint administration of TC-G 1008 and CGP 37849 (*ip*). Locomotor activity was measured with the Any Maze video tracking software (Stoelting, USA) as the overall distance travelled.

Table 4. Locomotor activity results for the effect of joint administration of TC-G 1008 and NBQX (*ip*). Locomotor activity was measured with the Any Maze video tracking software (Stoelting, USA) as the overall distance travelled.

Table 5. Locomotor activity results for the effect of joint administration of TC-G 1008 and CX 614 (*ip*). Locomotor activity was measured with the Any Maze video tracking software (Stoelting, USA) as the overall distance travelled.

Table 6. Locomotor activity results for the effect of joint administration of TC-G 1008 and picrotoxin (*ip*). Locomotor activity was measured with the Any Maze video tracking software (Stoelting, USA) as the overall distance travelled.

Table 7. Locomotor activity results for the effect of joint administration of TC-G 1008 and muscimol (*ip*). Locomotor activity was measured with the Any Maze video tracking software (Stoelting, USA) as the overall distance travelled.

Table 8. Locomotor activity results for the effect of joint administration of TC-G 1008 and R-baclofen (*ip*). Locomotor activity was measured with the Any Maze video tracking software (Stoelting, USA) as the overall distance travelled.

Table 9. Locomotor activity results for the effect of joint administration of TC-G 1008 and SCH 50911 (*ip*). Locomotor activity was measured with the Any Maze video tracking software (Stoelting, USA) as the overall distance travelled.

Table 10. Locomotor activity results for the effect of low-zinc diet on the antidepressant-like effects of TC-G 1008. Locomotor activity was measured with the Any Maze video tracking software (Stoelting, USA) as the overall distance travelled. ZnA – control diet, ZnD – low-zinc diet.
